# Bibliometric Analysis of Publications on Prenatal Genetic Screening Using Cell-Free DNA

**DOI:** 10.7759/cureus.80017

**Published:** 2025-03-04

**Authors:** Evren Koçbulut, Ahmet Kurt, Dilara Sarikaya Kurt, Harun Egemen Tolunay

**Affiliations:** 1 Obstetrics and Gynaecology, Private Clinic, Ankara, TUR; 2 Obstetrics and Gynaecology, Ankara Etlik City Hospital, Ankara, TUR; 3 Perinatology, Private Clinic, Ankara, TUR

**Keywords:** bibliometric analysis, cell-free dna, noninvasive prenatal testing (nipt), prenatal genetic screening, publications

## Abstract

Prenatal genetic testing plays a vital role in the early detection of fetal chromosomal abnormalities, with cell-free DNA (cfDNA) testing emerging as a highly accurate noninvasive screening method. By analyzing fetal DNA fragments in maternal plasma, cfDNA-based tests have significantly improved the detection of conditions such as trisomies.

Bibliometric analysis is a quantitative research method used to evaluate publication trends, citation patterns, and research impact within a specific scientific field. By analyzing bibliographic data, it provides insights into scholarly productivity, influential works, and collaboration networks, helping to identify key developments and emerging research areas.

This bibliometric analysis provides a comprehensive overview of publication patterns and impacts in the field of prenatal genetic screening with cfDNA between 1991 and 2024.

This study examined research articles and reviews on cfDNA used in prenatal genetic screening in English-language literature published between 1991 and May 1, 2024. The study analyzed various parameters, including top published institutions, countries, journals, citations, and funding organizations. Data was collected from the Web of Science bibliometric database using Medical Subject Headings (MeSH) keywords related to the research topic and gathered for analyzing publications and citations. VOSviewer (Centre for Science and Technology Studies (CWTS), Leiden University, the Netherlands) was used for co-authorship among top published organizations and countries and for keyword analyses.

The study included 2272 publications on cfDNA revealing a diverse range of topics, with chromosome disorders being the most common. The majority of publications in the literature were published in journals indexed in the Science Citation Index Expanded (SCIE). The top five countries in global research contributions were the United States, China, England, Italy, and the Netherlands. The top organizations/universities contributing to these publications included the Chinese University of Hong Kong, BGI Shenzhen, Baylor College of Medicine, and Tufts University. Keyword analysis revealed a vast array of keywords, with "prenatal diagnosis" dominating the discourse. Top journals on cfDNA publications include Prenatal Diagnosis (269 publications), Clinical Chemistry (78 publications), and the American Journal of Obstetrics and Gynecology (72 publications), making them the leading platforms for research dissemination in this field.

This bibliometric analysis highlights the growing impact of cfDNA-based prenatal genetic screening, revealing key contributors, influential studies, and research trends shaping the field. The findings underscore the significant role of cfDNA technology in improving noninvasive prenatal testing and guiding future research directions in this domain.

## Introduction and background

Prenatal genetic testing encompasses a range of screening and diagnostic procedures aimed at assessing fetal genetic conditions during pregnancy. These tests play a crucial role in the early detection of chromosomal abnormalities and genetic disorders, enabling informed clinical decision-making. Among these, cell-free DNA (cfDNA) testing has emerged as a groundbreaking noninvasive screening method that analyzes fetal DNA fragments circulating in maternal plasma. cfDNA-based screening has significantly improved the detection of conditions such as trisomies (e.g., Down syndrome) and certain monogenic disorders, offering high sensitivity and specificity compared to conventional prenatal screening techniques. Chromosomes are small "packages" in cells that contain genes, and if one of these pairs has an extra copy of a chromosome, it causes changes in body and brain development [1,2].

Down syndrome is the most common chromosome disorder in the United States, while other trisomy disorders include Edwards syndrome (trisomy 18) and Patau syndrome (trisomy 13). A cfDNA screening can be done as early as the 10th week of pregnancy [2]. Pregnant women at higher risk of having a baby with a chromosome disorder are recommended for this screening. Factors such as age, having another baby with a chromosome disorder, abnormal fetal ultrasound results, and other prenatal test results can increase the risk of having a baby with Down syndrome or other trisomy disorders [3,4].

Prenatal screening tests began in the 1970s by evaluating maternal age and progressed to analyzing cfDNA in maternal blood. Despite its success in detecting common aneuploidies and trisomy 21, cfDNA is currently out of use due to factors affecting results and false positivity rates, necessitating invasive tests for positive results [3]. Prenatal screening tests identify pregnant women at high risk for chromosomal aneuploidy in early pregnancy, advising them based on their existing risks and preferences. Traditional tests combine maternal blood biomarkers with ultrasound findings, while cfDNA analyzes fetal DNA fragments from maternal blood. The selection of cfDNA testing population, benefits, costs, limitations, and clinical advantages are currently debated [4].

Researchers suggest that impaired placentation may alter cfDNA levels in patients with obstetric complications, but studies have conflicting results, and few prospectively evaluated nonreportable cfDNA screening outcomes, focusing on fetal fraction or maternal characteristics [5-7]. A remarkable study assessed the outcomes of pregnancies with nonreportable prenatal cfDNA screening results in a cohort of patients with complete genetic and obstetrical outcomes. It involved 25,199 pregnant individuals and 20,194 participants. The primary outcome was a risk for adverse obstetrical and perinatal outcomes, including aneuploidy, preterm birth, preeclampsia, small for gestational age, and stillbirth. The study concluded that nonreportable cfDNA results increase the risk [8]. Nonreportable results in most series can be due to inadequate fetal DNA, inability to interpret sequencing results, and inconclusive data [9,10].

Existing studies have primarily focused on the technical aspects and clinical applications of cfDNA testing; however, a comprehensive bibliometric analysis that maps global research trends, identifies influential contributions, and highlights key areas for future investigation is still lacking. Therefore, this study aims to systematically analyze the evolution of cfDNA-based prenatal genetic screening research, providing insights into publication trends, leading contributors, and emerging research directions.

In parallel, bibliometric analysis is a valuable method for systematically evaluating publication trends, research impact, and collaboration networks within a specific scientific domain. By analyzing citation patterns, publication outputs, and thematic developments, bibliometric studies provide insights into the evolution and research priorities of a given field. This study employs bibliometric analysis to assess global research trends in cfDNA-based prenatal genetic screening, identifying key contributors, influential studies, and emerging topics in this rapidly advancing discipline.

Materials and methods

In this retrospective bibliometric study, research articles and reviews on cfDNA used in prenatal genetic screening in the English-language literature published between 1991 and May 1, 2024, were examined in detail. During this comprehensive review process, information on such publications in the literature was meticulously analyzed. The study included many bibliometric parameters, from identifying the institutions and countries with the highest number of publications to determining the journals that publish the most articles in this field, from identifying the most cited publications to analyzing the subject content of the citations. We also focused on important information such as the organizations that fund research in this field.

Within the scope of this study, we aimed to collect data from the Web of Science (WoS) bibliometric database, including Medical Subject Headings (MeSH) keywords "Prenatal Testing", "Noninvasive Prenatal Screening", "Noninvasive Prenatal Diagnosis", "Prenatal Cell-Free DNA Screening", and "Prenatal cfDNA Screening". By Boolean methodology, the search was performed using the OR operator in the title. English was chosen as the search language. With this method, we aimed to systematically search the relevant literature and obtain a comprehensive dataset.

Bibliometric analysis

This study primarily employed descriptive bibliometric analysis without the use of inferential statistical tests. The study gathered data from the WoS database, including titles, document types, publication years, affiliations, keywords, publishing journals, abstracts, and citations, which were saved as TXT files and retrieved into Microsoft Office Excel 2019 (Microsoft Corporation, Redmond, Washington, United States). Microsoft Excel 2019 was utilized for analyzing publications, citations, and H-index, while VOSviewer (Centre for Science and Technology Studies (CWTS), Leiden University, the Netherlands) was used to create co-occurrence maps of countries, authors, and keywords [11-15]. Graphs and trend visualizations were created using Microsoft Excel 2019 and VOSviewer to provide a comprehensive overview of research dynamics in the field of cfDNA-based prenatal screening.

Inclusion and exclusion criteria

This study included research articles and reviews on cfDNA-based prenatal genetic screening published in English between 1991 and May 1, 2024. Studies were selected from the WoS bibliometric database using predefined MeSH keywords related to cfDNA and prenatal screening. Only articles indexed in the Science Citation Index Expanded (SCIE), Social Sciences Citation Index (SSCI), and Emerging Sources Citation Index (ESCI) were considered to ensure the inclusion of high-quality research.

The exclusion criteria were as follows: conference abstracts, book chapters, editorials, commentaries, and letters to the editor were excluded to maintain the focus on peer-reviewed research. Additionally, studies not written in English or lacking relevant bibliometric data (such as missing citation information) were omitted from the analysis.

## Review

Main features

According to the inclusion criteria, 2272 publications were reached. Of the publications, 1919 (84.463%) were articles and 353 (15.537%) were review articles. The majority of publications (90.801%) were published in journals indexed in SCIE, with ESCI representing 7.482%, SSCI 3.741%, the Conference Proceedings Citation Index-Science (CPCI-S) 2.905%, the Book Citation Index-Science (BKCI-S) and Book Citation Index-Social Sciences and Humanities (BKCI-SSH) 1.849% and 0.132%, respectively, and the Arts and Humanities Citation Index (AHCI) 0.088% (some journals were indexed in more than one field). Other significant contributions include the SSCI, CPCI-S, BKCI-S, and BKCI-SSH.

Of the 2272 publications included in our study, 1223 (53.829%) were published in the open-access model. Among these, 514 publications (22.623%) were gold access, 250 publications (11.004%) were gold hybrid, and 302 publications (13.292%) had free readable content. There were 676 publications (29.754%) in the green published category citation topics.

Table 1 details the various citation topics and their prevalence in the dataset. In total, there are 58 different topics, reflecting the diversity and scope of the field. The most common topic is chromosome disorders (83.231%), which probably represents an important area of genetic research. Other topics such as lung cancer (2.465%) and obstetrics and gynecology (2.245%) were also prominent topics.

**Table 1:** Citation topics meso

Citation topics meso	n, %
Chromosome disorders	1891 (83.231%)
Lung cancer	56 (2.465%)
Obstetrics and gynecology	51 (2.245%)
Hematologic diseases	37 (1.629%)
Reproductive biology	29 (1.276%)
Genome studies	22 (0.968%)
Blood disorders	14 (0.616%)
Molecular and cell biology: genetics	13 (0.572%)
Micro and long noncoding RNA	9 (0.396%)
Adrenal disorders	8 (0.352%)
Extracellular matrix and cell differentiation	7 (0.308%)
Musculoskeletal disorders	6 (0.264%)
Birth defects	6 (0.264%)
Diabetes	5 (0.220%)
Microfluidic devices and superhydrophobicity	5 (0.220%)
Autism and development disorders	4 (0.176%)
Throat and voice disorders	4 (0.176%)
Tissue barriers	4 (0.176%)
Molecular and cell biology: cancer, autophagy, and apoptosis	4 (0.176%)
Cardiology: general	4 (0.176%)
Biosensors	4 (0.176%)
Mass spectrometry	4 (0.176%)
Molecular and cell biology: cancer and development	3 (0.132%)
Virology: identification and sequencing	3 (0.132%)
Abdominal surgery	3 (0.132%)

Table 2 shows the most common citation topics, with nuchal translucency accounting for 80.106% of the total. Circulating tumor cells is the most common topic, followed by DiGeorge syndrome and preeclampsia. Turner syndrome, alloimmunization, and sickle cell disease follow closely, followed by BRCA1 and preterm labor. Immune thrombocytopenia and oocyte are the most common topics, followed by Prader-Willi syndrome and congenital adrenal hyperplasia. Other topics like DNA methylation, population genetics, microRNAs, Duchenne muscular dystrophy, islets, HLA-G, fertility preservation, and twin-twin transfusion syndrome have fewer records, ranging from 0.220% to 5%.

**Table 2:** Citation topics micro The table shows 25 out of 103 entries; 36 record(s) (1.585%) do not contain data in the field being analyzed.

Citation topics micro	n, %
Nuchal translucency	1820 (80.106%)
Circulating tumor cells	55 (2.421%)
DiGeorge syndrome	35 (1.540%)
Preeclampsia	29 (1.276%)
Turner syndrome	24 (1.056%)
Alloimmunization	20 (0.880%)
Sickle cell disease	16 (0.704%)
BRCA1	15 (0.660%)
Preterm labor	12 (0.528%)
Immune thrombocytopenic purpura	10 (0.440%)
Oocyte	10 (0.440%)
Prader-Willi syndrome	9 (0.396%)
Congenital adrenal hyperplasia	8 (0.352%)
DNA methylation	6 (0.264%)
Population genetics	5 (0.220%)
MicroRNAs	5 (0.220%)
Duchenne muscular dystrophy	5 (0.220%)
Islets	5 (0.220%)
HLA-G	5 (0.220%)
Fertility preservation	5 (0.220%)
Twin-twin transfusion syndrome	5 (0.220%)
Craniosynostosis	4 (0.176%)
Down syndrome	4 (0.176%)
Exosomes	4 (0.176%)
Tracheal stenosis	4 (0.176%)

Publication trends

The historical distribution of publications was analyzed. According to the distribution of publications by year, 2019 and 2020 had the highest number of publications, followed by 2021 and 2022. However, a decrease was observed in 2023 and 2024. In the 1990s and early 2000s, the number of publications was quite low, but increased over time and showed a more steady upward trend towards the 2010s.

Top cited articles and citations by years

**Figure 1:**
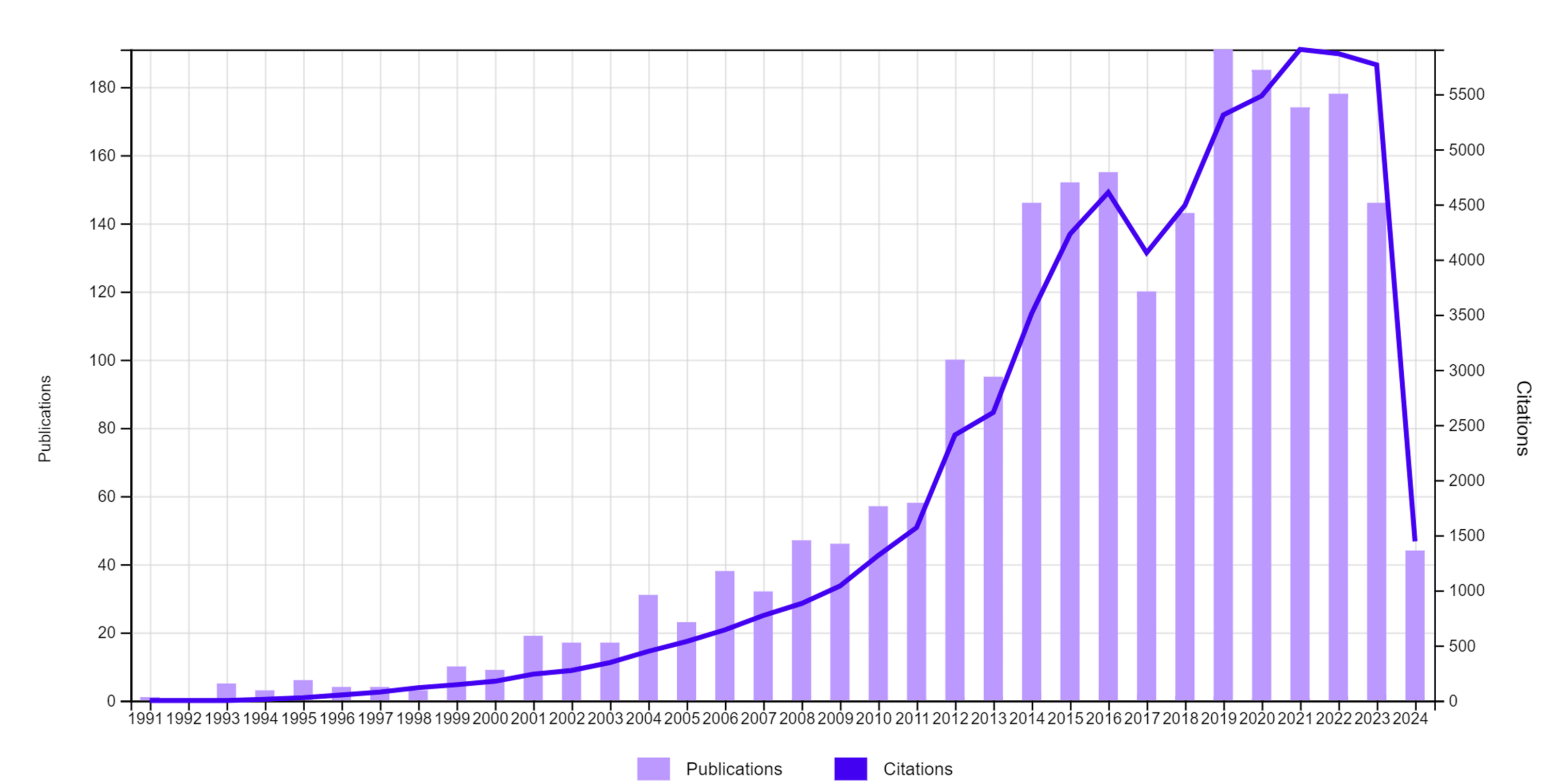
Top cited articles and citations by years

These studies were cited 64,803 times in total and 41,554 times without including self-citations. On average, each study was cited 28.52 times. The H-index was 110. The number of citations according to the most cited years was as follows: 5311 in 2019, 5483 in 2020, 5906 in 2021, 5878 in 2022, and 5857 in 2023 (Figure 1). 

The top cited articles are summarized in Table 3. These papers represent the most important research in the field of noninvasive prenatal diagnosis. These top five studies are among the pioneers of technological and methodological innovations that enable the detection of fetal genetic abnormalities from maternal blood samples. In particular, advanced technological approaches such as microfluidic systems for cfDNA analysis, digital polymerase chain reaction (PCR), and large-scale sequencing technologies are enabling sensitive and specific detection of fetal DNA. These studies also include methods that have been successfully applied to noninvasive prenatal screening for common genetic disorders such as Down syndrome, Edwards syndrome, and Patau syndrome. Furthermore, the circulation of fetal cells in the mother's circulation is another important focus of research in this field. These papers represent revolutionary advances in prenatal diagnosis and a milestone in enabling expectant parents to make informed decisions. The top three most cited papers in the table stand out as important references. The study by Lo and colleagues, published in 1998, provided important clues for noninvasive prenatal diagnosis by quantitative analysis of fetal DNA in maternal plasma and serum and received a total of 1251 citations. In second place, the 1996 article by Bianchi et al. focused on the discovery of male fetal progenitor cells that can persist in the mother's blood for long periods of time and received 944 citations. In third place, the 2011 study by Kinde et al. introduced a method to detect rare mutations by large-scale parallel sequencing and received 831 citations in total.

**Table 3:** Top cited articles

Title	Authors	Source title	Publication year	DOI	Total citations	Average per year
Quantitative analysis of fetal DNA in maternal plasma and serum: implications for noninvasive prenatal diagnosis	Lo et al. [16]	American Journal of Human Genetics	1998	10.1086/301800	1251	46.33
Male fetal progenitor cells persist in maternal blood for as long as 27 years postpartum	Bianchi et al. [17]	Proceedings of the National Academy of Sciences of the United States of America	1996	10.1073/pnas.93.2.705	944	32.55
Detection and quantification of rare mutations with massively parallel sequencing	Kinde et al. [18]	Proceedings of the National Academy of Sciences of the United States of America	2011	10.1073/pnas.1105422108	831	59.36
Noninvasive diagnosis of fetal aneuploidy by shotgun sequencing DNA from maternal blood	Fan et al. [19]	Proceedings of the National Academy of Sciences of the United States of America	2008	10.1073/pnas.0808319105	781	45.94
Maternal plasma DNA sequencing reveals the genome-wide genetic and mutational profile of the fetus	Lo et al. [20]	Science Translational Medicine	2010	10.1126/scitranslmed.3001720	757	50.47
Detection and characterization of placental microRNAs in maternal plasma	Chim et al. [21]	Clinical Chemistry	2008	10.1373/clinchem.2007.097972	681	40.06
Noninvasive prenatal diagnosis of fetal chromosomal aneuploidy by massively parallel genomic sequencing of DNA in maternal plasma	Chiu et al. [22]	Proceedings of the National Academy of Sciences of the United States of America	2008	10.1073/pnas.0810641105	677	39.82
Genome-wide fetal aneuploidy detection by maternal plasma DNA sequencing	Bianchi et al. [23]	Obstetrics and Gynecology	2012	10.1097/AOG.0b013e31824fb482	488	37.54
Plasma DNA tissue mapping by genome-wide methylation sequencing for noninvasive prenatal, cancer, and transplantation assessments	Sun et al. [24]	Proceedings of the National Academy of Sciences of the United States of America	2015	10.1073/pnas.1508736112	461	46.1
DNA sequencing versus standard prenatal aneuploidy screening	Bianchi et al. [25]	New England Journal of Medicine	2014	10.1056/NEJMoa1311037	461	41.91
Noninvasive prenatal screening for fetal aneuploidy, 2016 update: a position statement of the American College of Medical Genetics and Genomics	Gregg et al. [26]	Genetics in Medicine	2016	10.1038/gim.2016.97	456	50.67
Size distributions of maternal and fetal DNA in maternal plasma	Chan et al. [27]	Clinical Chemistry	2004	10.1373/clinchem.2003.024893	442	21.05
Analysis of cell-free DNA in maternal blood in screening for aneuploidies: updated meta-analysis	Gil et al. [28]	Ultrasound in Obstetrics and Gynecology	2017	10.1002/uog.17484	424	53
Non-Invasive Chromosomal Evaluation (NICE) study: results of a multicenter prospective cohort study for detection of fetal trisomy 21 and trisomy 18	Norton et al. [29]	American Journal of Obstetrics and Gynecology	2012	10.1016/j.ajog.2012.05.021	416	32
DNA sequencing of maternal plasma reliably identifies trisomy 18 and trisomy 13 as well as Down syndrome: an international collaborative study	Palomaki et al. [30]	Genetics in Medicine	2012	10.1038/gim.2011.73	408	31.38
Analysis of cell-free DNA in maternal blood in screening for fetal aneuploidies: updated meta-analysis	Gil et al. [31]	Ultrasound in Obstetrics and Gynecology	2015	10.1002/uog.14791	401	40.1
Circulating cytokines, chemokines and adhesion molecules in normal pregnancy and preeclampsia determined by multiplex suspension array	Szarka et al. [32]	BMC Immunology	2010	10.1186/1471-2172-11-59	396	26.4
Cancer genome scanning in plasma: detection of tumor-associated copy number aberrations, single-nucleotide variants, and tumoral heterogeneity by massively parallel sequencing	Chan et al. [33]	Clinical Chemistry	2013	10.1373/clinchem.2012.196014	374	31.17
Noninvasive detection of fetal trisomy 21 by sequencing of DNA in maternal blood: a study in a clinical setting	Ehrich et al. [34]	American Journal of Obstetrics and Gynecology	2011	10.1016/j.ajog.2010.12.060	372	26.57
Microfluidics digital PCR reveals a higher than expected fraction of fetal DNA in maternal plasma	Lun et al. [35]	Clinical Chemistry	2008	10.1373/clinchem.2008.111385	340	20
Digital PCR for the molecular detection of fetal chromosomal aneuploidy	Lo et al. [36]	Proceedings of the National Academy of Sciences of the United States of America	2007	10.1073/pnas.0705765104	334	18.56
Noninvasive prenatal testing for fetal trisomies in a routinely screened first-trimester population	Nicolaides et al. [37]	American Journal of Obstetrics and Gynecology	2012	10.1016/j.ajog.2012.08.033	294	22.62
Noninvasive whole-genome sequencing of a human fetus	Kitzman et al. [38]	Science Translational Medicine	2012	10.1126/scitranslmed.3004323	290	22.31
Plasma placental RNA allelic ratio permits noninvasive prenatal chromosomal aneuploidy detection	Lo et al. [39]	Nature Medicine	2007	10.1038/nm1530	289	16.06
mRNA of placental origin is readily detectable in maternal plasma	Ng et al. [40]	Proceedings of the National Academy of Sciences of the United States of America	2003	10.1073/pnas.0637450100	288	13.09

The top five articles listed in the table focused on the following topics: The first is "Quantitative analysis of fetal DNA in maternal plasma and serum: implications for noninvasive prenatal diagnosis" (1998) [16]. Quantitative analysis of fetal DNA in maternal plasma and serum has provided an important starting point for noninvasive prenatal diagnosis. This work has contributed to the development of techniques used in prenatal diagnosis.

The second is "Male fetal progenitor cells persist in maternal blood for as long as 27 years postpartum" (1996) [17]. This study investigated the persistence of male fetal progenitor cells in maternal blood decades after pregnancy. By analyzing blood samples from women who had previously given birth to male offspring, the researchers detected male DNA in maternal blood up to 27 years postpartum. These findings suggest the long-term presence of fetal cells, known as microchimerism, which could have implications for immune tolerance and prenatal diagnostics.

The third is "Detection and quantification of rare mutations with massively parallel sequencing" (2011) [18]. This is a study on the detection and quantification of rare mutations with massively parallel sequencing. This paper is considered an important step in the development of methods used in the diagnosis and treatment of genetic diseases.

The fourth is "Noninvasive prenatal diagnosis of fetal chromosomal aneuploidy by massively parallel genomic sequencing of DNA in maternal plasma" (2008) [22]. This study provides a noninvasive prenatal diagnosis of fetal chromosomal aneuploidies by large-scale genomic sequencing of maternal plasma DNA. This is a revolutionary advance in the field of prenatal diagnosis.

The fifth is "Noninvasive prenatal screening for fetal aneuploidy, 2016 update: a position statement of the American College of Medical Genetics and Genomics" (2016) [26]. The 2016 update of the American College of Medical Genetics and Genomics states a position for noninvasive prenatal screening of fetal aneuploidies. This work is important to provide guidance in clinical practice.

Global funding agencies

**Figure 2:**
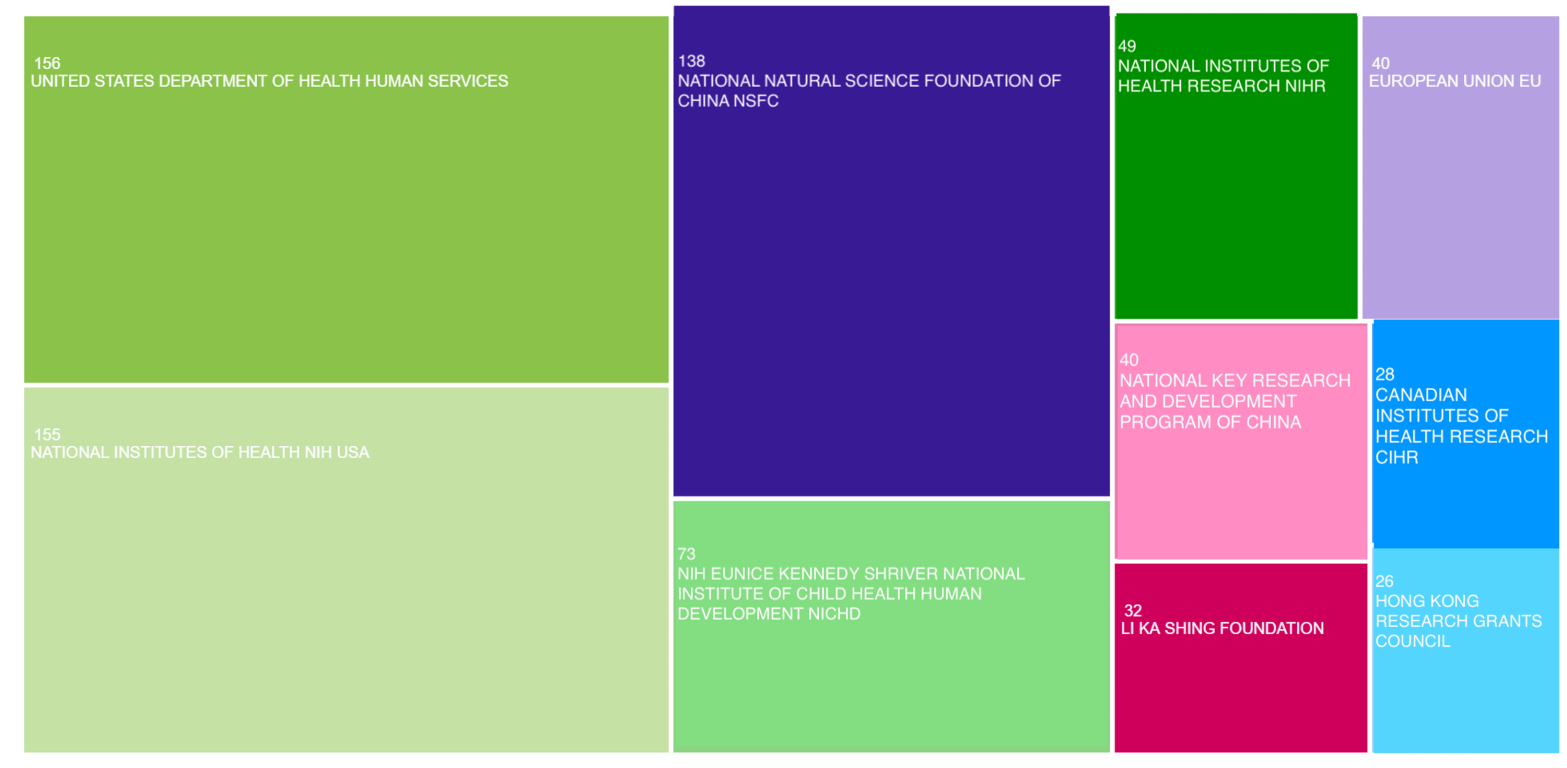
Global funding agencies

The United States Department of Health and Human Services led the list of funding agencies with 156 records (6.866%), supporting various health and human services initiatives. The National Institutes of Health (NIH) USA followed closely with 155 records (6.822%), a major player in scientific research worldwide. The National Natural Science Foundation of China (NSFC) was a key organization supporting scientific research and development activities in China. The NIH Eunice Kennedy Shriver National Institute of Child Health and Human Development (NICHD) was a leading research institution in the United States. The European Union represented 40 records (1.761%), while China's National Key Research and Development Program had 40 records (1.761%). Other notable institutions included the Hong Kong Research Grants Council, the Canadian Institutes of Health Research, and the Great Ormond Street Hospital Children's Charity (Figure 2).

Group authors

The Dutch Non-Invasive Prenatal Testing (NIPT) Consortium had the most records (0.132%), followed by the Global Expanded NIPT Consortium and Japan NIPT Consortium (0.088%). Other group authors included the American College of Medical Genetics and Genomics (ACMG) Board of Directors, ACMG Lab Quality Assurance Committee, and ACMG Professional Practice Guidelines (0.044%). Other group authors include Collaborative Sequenage Haut Debit, Copenhagen Pregnancy Loss (COPL) Consortium, Gencounsel Study, Genetics Committee, H Consortium, Japan NIPT Consortium, Maternal Blood Is Source Accurately, NIPT MAP Study Group, Obstetrix Collaborative Research Network, Prenatal Testing PAG Coalition, and SAFE 21 Study Group (0.044%).

Top published and top cited countries

**Figure 3:**
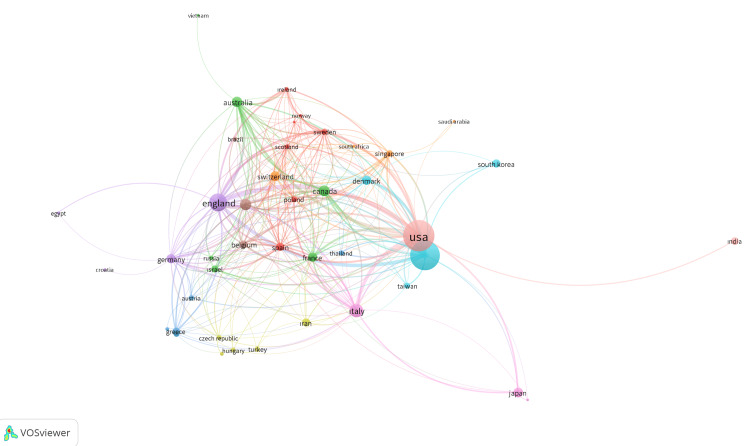
Top published and top cited countries

Authors from 76 countries contributed to these publications. The data visualization of countries with five or more publications with VOSviewer is visualized in Figure 3. The top 10 countries in global research contributions include the United States, China, England, Italy, the Netherlands, Canada, Australia, Japan, Switzerland, and Denmark. The United States had the most significant scientific output, with 655 documents and 28,028 citations. China had 602 documents and 17,902 citations. England had 212 documents and 10,582 citations. Italy had 125 papers and 2760 citations. The Netherlands had 93 papers and 3801 citations. Canada had 79 papers and 1335 citations. Australia had 71 papers and 1420 citations (Table 4).

**Table 4:** Number of scientific documents, citations, and total linking power of countries

Country	Number of documents	Number of citations	Total link strength
United States	655	28,028	298
People's Republic of China	602	17,902	168
England	212	10,582	201
Italy	125	2760	103
Netherlands	93	3801	89
Canada	79	1335	69
Australia	71	1420	95
Japan	67	1469	21
Switzerland	66	1948	51
Denmark	61	1518	49
Germany	61	1168	72
France	60	1617	52
Spain	49	643	82
South Korea	46	620	8
Iran	44	355	16
Belgium	43	953	74
Greece	40	901	26
India	40	367	4
Israel	38	588	33
Singapore	30	648	48
Poland	25	383	49
Taiwan	25	430	15
Cyprus	24	738	24
Sweden	24	868	74
Turkey	23	288	5
Austria	22	431	17
Thailand	21	677	16
Czech Republic	17	144	18
Hungary	16	845	9
Brazil	14	210	13
Russia	14	134	14
Scotland	14	205	41
Slovakia	12	102	3
Romania	11	58	3
Ireland	10	134	39
Egypt	9	426	4
Croatia	7	103	1
Norway	6	113	19
South Africa	6	36	15
Finland	5	66	13
Saudi Arabia	5	27	2
Vietnam	5	10	1
Indonesia	5	43	4

Top published and top cited organizations/universities

**Figure 4:**
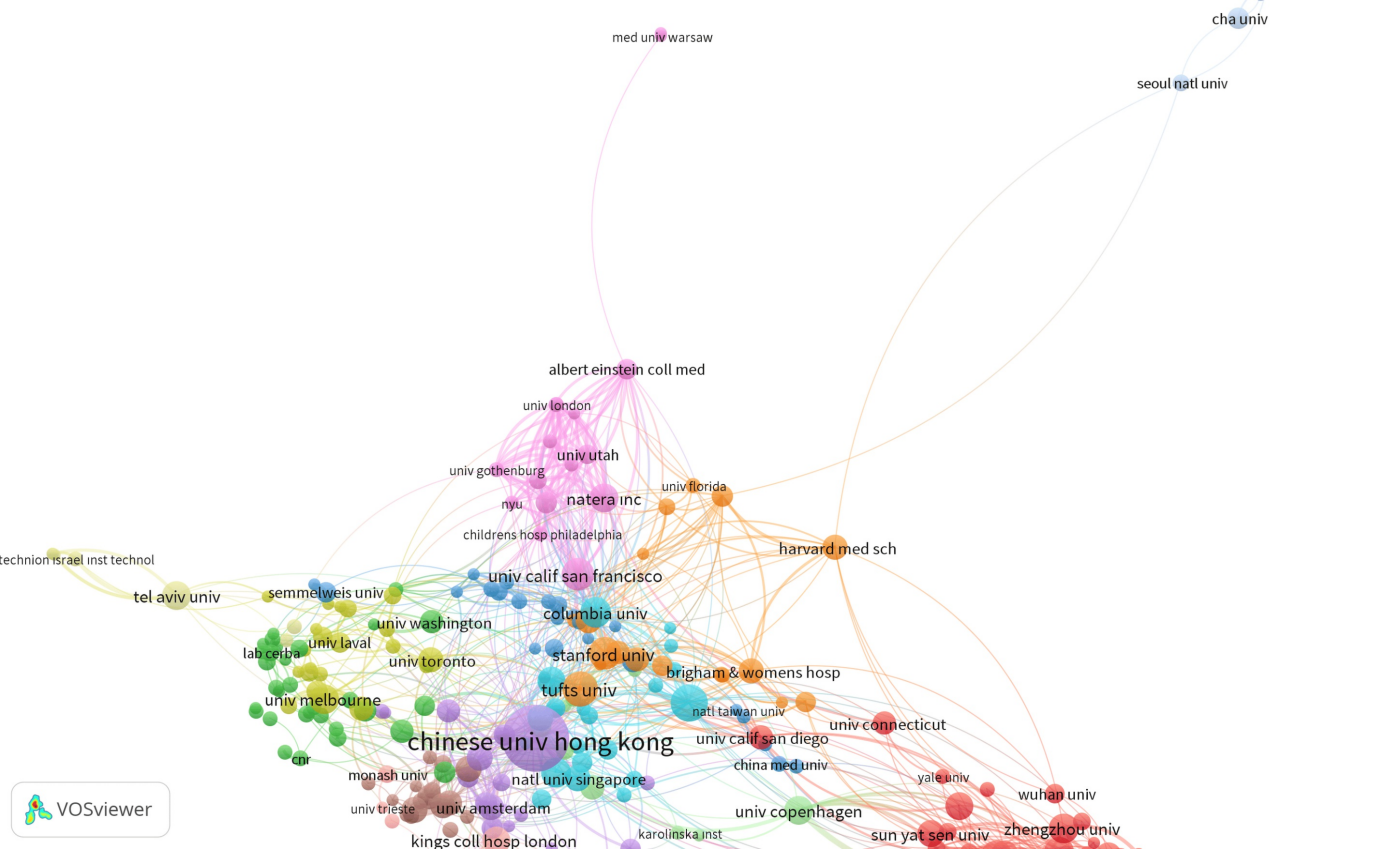
Top published and top cited organizations/universities

There were 2647 authors from different organizations/universities who contributed to these field publications. Organizations/universities with the most publications were summarized and visualized by VOSviewer in Figure 4.

Table 5 presents the number of scientific documents, citations, and total linking power of top published organizations and universities. The Chinese University of Hong Kong had the highest number of documents, with 13,592 citations and a total link strength of 80. BGI Shenzhen had the second highest number of documents, with 1164 citations and a total link strength of 98. Baylor College of Medicine had the third highest number of documents, with 1480 citations and a total link strength of 62. Tufts University had the fourth highest number of documents, with 3223 citations and a total link strength of 47.

**Table 5:** Number of scientific documents, citations, and total linking power of top published organizations/universities UCL: University College London

Organizations/universities	Number of documents	Number of citations	Total link strength
Chinese University of Hong Kong	138	13,592	80
BGI Shenzhen	47	1164	98
Baylor College of Medicine	42	1480	62
Tufts University	39	3223	47
Nanjing Medical University	38	707	30
Stanford University	33	2580	30
University of California, San Francisco	33	1584	103
Guangzhou Medical University	31	444	46
Columbia University	29	1417	68
Zhengzhou University	28	294	37
Shanghai Jiao Tong University	27	457	40
University of Basel	27	964	10
University of Melbourne	27	674	57
Natera Inc.	26	1278	82
Tel Aviv University	26	290	37
University of Copenhagen	26	792	51
Brown University	25	1446	46
King's College Hospital London	25	1915	26
Sichuan University	24	345	13
University of Athens	24	483	18
Southern Medical University	23	220	40
Sun Yat Sen University	23	285	29
Fudan University	22	343	31
Mayo Clinic	22	283	24
National University of Singapore	22	392	29
Leiden University	21	949	38
Sequenom Inc.	21	3483	41
UCL	21	626	42
University of Amsterdam	21	936	46
University College London Hospital NHS Foundation Trust	21	961	54
Brigham and Women's Hospital	20	949	34
Great Ormond Street Hospital for Sick Children	20	827	43
Harvard Medical School	20	608	28
UCL Institute of Child Health	20	915	44
University of Toronto	20	254	27

Keyword analysis

A total of 3077 keywords were used in these publications and 229 of them were used five times or more.

**Figure 5:**
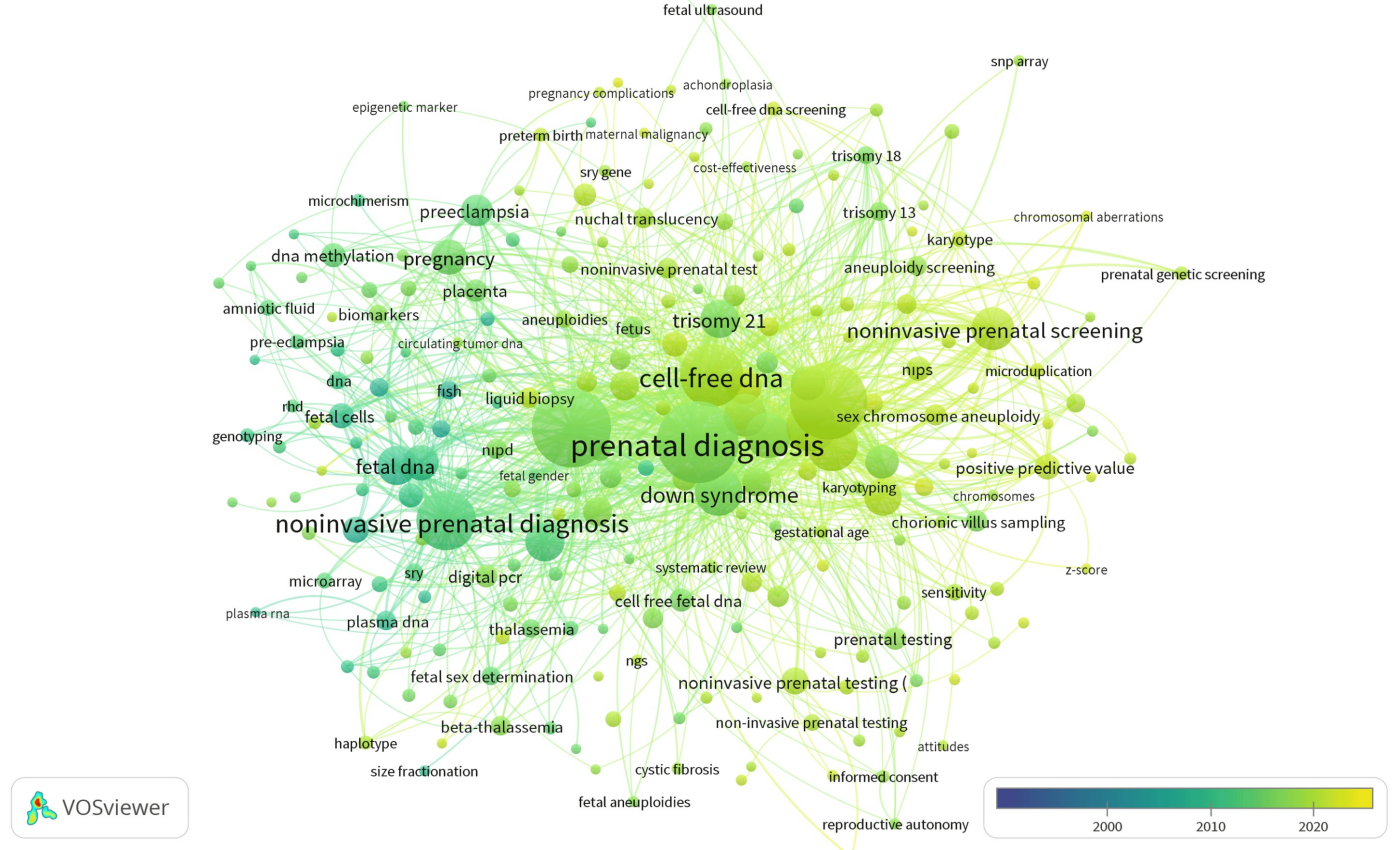
Keyword analysis

The keyword analysis with VOSviewer revealed a vast array of keywords in publications, with prenatal diagnosis dominating the discourse with 294 mentions and a strong total link strength of 741. Cell-free fetal DNA was a prominent focus, with 270 occurrences and a link strength of 642. Noninvasive prenatal testing was noted 261 times with a total link strength of 652. Cell-free DNA was cited 172 times with a total link strength of 489. Noninvasive prenatal diagnosis was cited 153 times and yielded a total link strength of 340. NIPT was cited 115 times, showcasing its widespread adoption and recognition. Prenatal screening was mentioned 109 times with a total link strength of 372. Keywords like "Down syndrome", "aneuploidy", and "preeclampsia" also contributed significantly to the discourse (Table 6). Figure 5 is a visualization of the most preferred keywords according to year periods.

**Table 6:** Analysis of keywords: number of occurrences and connection strength

Keyword	Number of occurrences	Total link strength
Prenatal diagnosis	294	741
Cell-free fetal DNA	270	642
Noninvasive prenatal testing	261	652
Cell-free DNA	172	489
Noninvasive prenatal diagnosis	153	340
NIPT	115	321
Prenatal screening	109	372
Down syndrome	100	327
Aneuploidy	91	310
Non-invasive prenatal testing	82	234
Noninvasive prenatal screening	81	225
Non-invasive prenatal diagnosis	68	151
Fetal DNA	67	142
Trisomy 21	64	212
Genetic counseling	60	161
Fetal fraction	59	148
Maternal plasma	56	139
Pregnancy	56	129
Trisomy	51	187
Amniocentesis	50	135
Preeclampsia	45	107
Next-generation sequencing	38	127
Screening	38	135
cffDNA	36	99
Noninvasive prenatal testing (NIPT)	33	56
Maternal blood	31	65
cfDNA	29	104
Fetal cells	29	71
Positive predictive value	29	96
DNA methylation	27	58
Prenatal	27	80
Real-time PCR	27	51
NIPS	26	74
Cell free fetal DNA	25	55

Top journals on cfDNA publications

Table 7 lists several journals that frequently publish articles on cfDNA. Prenatal Diagnosis is the most prolific journal, with 269 documents and 7964 citations. Clinical Chemistry follows closely with 78 documents and 6428 citations. The American Journal of Obstetrics and Gynecology ranks third with 72 documents and 4092 citations. Other notable journals include PLOS One, Fetal Diagnosis and Therapy, and the Journal of Maternal-Fetal and Neonatal Medicine. Genetics in Medicine, Obstetrics and Gynecology, Molecular Cytogenetics, and the Journal of Genetic Counseling also contribute significantly to the discourse on cfDNA.

**Table 7:** List of journals that most frequently publish articles on cell-free DNA

Journal	Number of documents	Number of citations
Prenatal Diagnosis	269	7964
Clinical Chemistry	78	6428
American Journal of Obstetrics and Gynecology	72	4092
PLOS One	58	1787
Fetal Diagnosis and Therapy	53	900
Journal of Maternal-Fetal and Neonatal Medicine	49	468
Genetics in Medicine	48	2621
Obstetrics and Gynecology	35	1959
Molecular Cytogenetics	32	251
Journal of Genetic Counseling	29	521
Journal of Obstetrics and Gynaecology Research	28	170
Molecular Genetics and Genomic Medicine	26	185
Frontiers in Genetics	25	109
BMC Pregnancy and Childbirth	24	333
Scientific Reports	24	271
European Journal of Obstetrics and Gynecology and Reproductive Biology	22	330
Expert Review of Molecular Diagnostics	22	249
Proceedings of the National Academy of Sciences of the United States of America	21	6130
Clinica Chimica Acta	18	376
Clinical Case Reports	18	61
Current Opinion in Obstetrics and Gynecology	18	308
Transfusion	18	973
Ultrasound in Obstetrics and Gynecology	17	874
Clinical Chemistry and Laboratory Medicine	16	218
Genes	16	194
Taiwanese Journal of Obstetrics and Gynecology	16	141
Diagnostics	15	91
BMC Medical Genomics	14	218
Journal of Fetal Medicine	14	38
International Journal of Molecular Sciences	14	196
Australian and New Zealand Journal of Obstetrics and Gynaecology	13	261
Human Reproduction	13	372
Journal of Assisted Reproduction and Genetics	13	76
European Journal of Human Genetics	12	269
Journal of Clinical Medicine	12	257
Placenta	12	707
American Journal of Obstetrics and Gynecology MFM	11	48
BJOG: An International Journal of Obstetrics and Gynaecology	11	488
Clinical Obstetrics and Gynecology	11	81
Journal of Molecular Diagnostics	11	83
Journal of Obstetrics and Gynaecology Canada	11	177
Best Practice and Research Clinical Obstetrics and Gynaecology	10	330
Clinical Biochemistry	10	481
Clinics in Laboratory Medicine	10	63
Circulating Nucleic Acids in Plasma and Serum IV	10	152

cfDNA, discovered by Mandel and Metais in 1948 [41], has shown promising potential in obstetrics and gynecology, particularly in prenatal diagnosis, genetic disease screening, fetal health monitoring, and early pregnancy complications detection. The use of cfDNA in noninvasive prenatal testing, such as analyzing fetal DNA in maternal blood, can provide early detection of genetic disorders and identify risks during pregnancy [15]. This research provides a comprehensive analysis of existing studies on cfDNA-based prenatal screening, highlighting research trends and key contributions that have shaped the field. By mapping the evolution of scientific knowledge, it offers insights that may guide future research and clinical applications in maternal and fetal health. This study presents a bibliometric analysis of cfDNA prenatal genetic screening published from 1991 to 2024, providing information on the evolution of research in this field, research trends, and global impact. There is a similar study previously published on this topic but no new study has already been published [15]. Gao et al.'s study analyzed global trends in cfDNA research in obstetrics and gynecology from 2017 to 2021. The analysis used 5038 pieces of literature and 527 articles from the WoS Core Collection. Key findings revealed cfDNA sequence analysis for noninvasive prenatal and genetic testing, as well as its application in neoplasm genetics and diagnosis. Their study identified five research hotspots: application of cfDNA in prenatal screening, assisted reproductive technology, preeclampsia, placental dysfunction, and fetal chromosomal abnormalities [15].

The analysis of prenatal genetic screening using cfDNA shows a steady increase in publications over the years, peaking in 2019 and 2020. However, there was a decline in publications in 2023 and 2024. While the exact reasons remain unclear, possible explanations may include technological advancements, increased clinical adoption, or shifts in research funding priorities. Additionally, external factors such as global events, policy changes, or economic conditions could have influenced publication trends. Further studies analyzing funding allocations and research priorities over time would be needed to confirm these potential influences. The peak in publications around 2019 and 2020 may have been fueled by significant advancements in cfDNA analysis techniques and sequencing technologies, while subsequent years may have seen fewer breakthroughs or innovations. The decline in publications in 2023 and 2024 may be counterintuitive given the increasing interest in the field, but it likely reflects the complex interplay of various factors shaping research trends in the field. Also, the year 2024 has not yet been completed.

Gao et al. focused on the period from 2017 to 2021 and identified the United States, China, and the United Kingdom as the top contributing countries, representing 61.74% of total publications. They noted that there are disparities in citations, with Chinese papers receiving significantly fewer citations compared to those from the United States and England [15]. In contrast, our study visualized the data using VOSviewer and highlighted countries with five or more publications. The United States was the leader in scientific output with 655 documents and 28,028 citations, followed by China, with 602 documents and 17,902 citations, and the United Kingdom, with 212 documents and 10,582 citations. They are among the leading contributors to research in this field. The development in many fields that China has witnessed in recent years, including the field of scientific research, played a role in this progress. Differences in findings between studies may be due to variations in time periods, the specific focus of the studies, and differences in historical perspectives. Additionally, discrepancies may arise from the choice of bibliographic databases, as some studies have utilized multiple sources, including PubMed, while our analysis is based solely on the WoS, which may influence the scope of retrieved publications.

The most common citation topic for cfDNA research is "chromosome disorders", which focuses on identifying and understanding genetic abnormalities in fetuses. These disorders, like Down syndrome, Edwards syndrome, and Patau syndrome, contribute to prenatal complications and developmental disorders [42]. Advancements in cfDNA-based prenatal genetic screening offer noninvasive and accurate detection. The research also includes topics like "lung cancer" and "obstetrics and gynecology", which highlight the interdisciplinary nature of cfDNA research. cfDNA-based liquid biopsy approaches have shown promise in early cancer detection and monitoring. The integration of cfDNA research with maternal health and reproductive medicine is also significant.

Gao et al.'s study on prenatal genetic screening and our study highlight the importance of prenatal diagnosis, cfDNA, and noninvasive prenatal testing in the early detection and management of genetic conditions such as Down syndrome and preeclampsia. Both studies emphasize the continuous advancements and shifting research priorities in prenatal genetic screening methodologies.

Prenatal diagnosis emerged as the most prevalent term, with 294 mentions and a link strength of 741, which is expected given its central role in identifying fetal genetic conditions and guiding clinical decision-making. Similarly, cfDNA was a prominent focus, with 270 mentions and a link strength of 642, reflecting its increasing adoption as a reliable method for noninvasive prenatal screening. The frequent mention of noninvasive prenatal diagnosis (153 times) highlights ongoing efforts to enhance diagnostic accuracy while minimizing risks associated with invasive procedures. Prenatal screening, with 109 mentions and a link strength of 372, underscores the widespread use of screening methods in early pregnancy to assess the likelihood of chromosomal abnormalities and other fetal conditions. Keywords like Down syndrome, aneuploidy, and preeclampsia also contributed to the discourse.

Gao et al.'s study highlighted influential journals like Prenatal Diagnosis, Ultrasound in Obstetrics and Gynecology, and American Journal of Obstetrics and Gynecology, focusing on clinical applications and diagnostic advancements. Our study found that the most prolific journal was Clinical Chemistry, with 269 documents and 7964 citations. Other journals included PLOS One, Fetal Diagnosis and Therapy, Journal of Maternal-Fetal and Neonatal Medicine, Genetics in Medicine, Obstetrics and Gynecology, Molecular Cytogenetics, and Journal of Genetic Counseling. Both studies highlight the critical role of cfDNA research in obstetrics and gynecology, but their analyses offer complementary perspectives.

It is possible to obtain information about journals that publish the most articles in a field using the bibliometric analysis method [12,14]. In our study, journals with the highest number of publications on cfDNA were examined. Prenatal Diagnosis journal was found to have the highest number of publications. As the official journal of the International Society for Prenatal Diagnosis, its aims and scope focus specifically on prenatal screening, genetic diagnosis, and related advancements, making it a primary platform for cfDNA research. The journal covers topics including prenatal molecular genetics, fetal therapy, fetal imaging, cytogenetics, genetic screening, and fetal development, which align closely with the scope of cfDNA research, explaining its prominence in the field.

Limitations

The study's data was sourced from the WoS bibliometric database, which may not include all relevant publications in this field. Other databases like PubMed or Scopus could have provided more insights. The search strategy was limited to English-language articles, potentially excluding valuable research in other languages. The analysis covered articles from 1991 to 2024, which may not fully capture the spectrum of research conducted in the field. The inclusion criteria, such as focusing on research articles and reviews, may have excluded other types of publications. The study did not assess the quality or methodological rigor of the included publications, potentially impacting the reliability and validity of the findings. The data extraction process may have errors or inconsistencies, potentially affecting the analysis's integrity. The interpretation of bibliometric data and keyword analysis is subjective and relies on the researcher's judgment, potentially introducing bias into the findings. VOSviewer analysis, while powerful, may not capture all relevant patterns or relationships, leading to potential oversights or misinterpretations.

## Conclusions

The study conducted a bibliometric analysis of cfDNA research in prenatal genetic screening from 1991 to 2024, examining publication trends, citation impact, leading contributors, and research dissemination across various journals. It found a significant increase in research output, particularly in 2019 and 2020, and emphasized the importance of prenatal diagnosis, cfDNA, and noninvasive prenatal testing in scholarly discourse. The United States, China, and England were identified as leading contributors to research publications, with institutions like the Chinese University of Hong Kong and BGI Shenzhen making significant impacts. Global funding agencies like the National Institutes of Health and the European Union supported cfDNA technologies in prenatal care. However, limitations like database biases and language restrictions suggest opportunities for future research to expand inclusivity and enhance the clinical applicability of cfDNA technologies worldwide.
